# Evidence for recent gene flow between north-eastern and south-eastern Madagascan poison frogs from a phylogeography of the *Mantella cowani *group

**DOI:** 10.1186/1742-9994-4-1

**Published:** 2007-01-06

**Authors:** Falitiana CE Rabemananjara, Ylenia Chiari, Olga Ravoahangimalala Ramilijaona, Miguel Vences

**Affiliations:** 1 Institute for Biodiversity and Ecosystem Dynamics, University of Amsterdam, Mauritskade 61, 1092 AD Amsterdam, The Netherlands; 2 Département de Biologie Animale, Université d'Antananarivo, BP, 906, 101 Antananarivo, Madagascar; 3 Zoological Museum, University of Amsterdam, Mauritskade 61, 1092 AD Amsterdam, The Netherlands; 4 Zoological Institute, Technical University of Braunschweig, Spielmannstr. 8, 38106, Braunschweig, Germany; 5 Department of Ecology and Evolutionary Biology, YIBS-Molecular Systematics and Conservation Genetics Lab., Yale University, 21 Sachem St., New Haven, CT, 06520-8105, USA

## Abstract

**Background:**

The genus *Mantella*, endemic poison frogs of Madagascar with 16 described species, are known in the field of international pet trade and entered under the CITES control for the last four years. The phylogeny and phylogeography of this genus have been recently subject of study for conservation purposes. Here we report on the studies of the phylogeography of the *Mantella cowani *group using a fragment of 453 bp of the mitochondrial cytochrome *b *gene from 195 individuals from 21 localities. This group is represented by five forms: *M. cowani*, a critically endangered species, a vulnerable species, *M. haraldmeieri*, and the non-threatened *M. baroni, M*. aff. *baroni*, and *M. nigricans*.

**Results:**

The Bayesian phylogenetic and haplotype network analyses revealed the presence of three separated haplotype clades: (1)* M. baroni, M*. aff. *baroni, M. nigricans*, and putative hybrids of *M. cowani *and *M. baroni*, (2) *M. cowani *and putative hybrids of *M. cowani *and *M. baroni*, and (3) *M. haraldmeieri*. The putative hybrids were collected from sites where *M. cowani *and *M. baroni *live in sympatry.

**Conclusion:**

These results suggest (a) a probable hybridization between *M. cowani *and *M. baroni*, (b) a lack of genetic differentiation between *M. baroni/M*. aff. *baroni *and *M. nigricans*, (c) evidence of recent gene-flow between the northern (*M. nigricans*), eastern (*M. baroni*), and south-eastern (*M*. aff. *baroni*) forms of distinct coloration, and (d) the existence of at least three units for conservation in the *Mantella cowani *group.

## Background

Madagascar, the fourth largest island of the world, is a hotspot for biodiversity that deserves the highest priority for conservation [1]. Currently, 233 amphibian species are known from this island [2]. All of the native taxa are endemic at the level of species, and all but one also at the level of genera [3-5]. Madagascar has so far been spared from one of the causes of the global decline of amphibians [5], the diseases due to dangerous pathogens, especially chytrid fungal infections [6-8]. However, the island is not safe from range contractions and extinctions due to habitat destruction and overexploitation of live specimens for the pet trade [5,9-14].

The poison frogs from Madagascar, *Mantella*, form one of the Malagasy endemic genera of amphibians. This genus contains 16 described species divided into six groups based on morphological and genetic criteria [15]. The genus holds the record in terms of Malagasy amphibians present in the pet trade (>230,000 individuals over 10 years 1994–2003) [16]. Species of *Mantella *are now included in the appendix II of the Convention on the International Trade in Endangered Species (CITES), and the commerce is thereby better regulated [16].

Genetic analyses to understand phylogeny and phylogeography of the genus *Mantella*, to solve problems in taxonomy, and to provide a basis for conservation actions to better protect species belonging to this genus are recent and are leading to more and more surprising new discoveries. Initial allozyme studies of a restricted number of taxa unveiled the existence of three lineages among the species from central-eastern rainforests: (1) *Mantella baroni *and *M. cowani*, (2) *M. madagascariensis *and *M. pulchra*, and (3) *M. aurantiaca, M. crocea *and *M. milotympanum *[17], and led to a revised classification of the genus [15]. The use of karyological methods provided some additional hints on the phylogeny of the genus [18], but it were mainly recent studies based on molecular analyses using mitochondrial and nuclear DNA sequences that provided progressively more precise information. In 2002, the use of the 16S rRNA marker in an attempt to elucidate the origin and evolution of the aposematic coloration of frogs in this genus permitted to find clades largely congruent [19] with the species groups defined by Vences et al. [15,17]: the *Mantella madagascariensis *group (*M. aurantiaca, M. crocea, M. madagascariensis, M. milotympanum, M. pulchra*), the *Mantella cowani *group (*M. baroni, M. cowani, M. haraldmeieri, M. nigricans*), the *Mantella bernhardi *group (*M. bernhardi*), the *Mantella betsileo *group (*M. betsileo, M*. sp. aff. *betsileo, M. expectata, M. viridis*) and the *Mantella laevigata *group (*M. laevigata*). Later, in 2004, the use of four different genetic markers (16S rRNA, 12S rRNA, cytochrome *b *and rhodopsin) confirmed this species-group division and revealed that *M. milotympanum *and *M. crocea *have a high degree of mitochondrial haplotype sharing, confirming doubts about the species validity of *M. milotympanum *and indicating independent evolution of bright orange pattern in *M. milotympanum *and *M. aurantiaca *[20]. Chiari et al. in 2004 [21] then used nuclear as well as mitochondrial DNA sequences (cytochrome *b*, Rag-1 and Rag-2) to investigate the evolution of coloration and the phylogeographic relationships of the species belonging to the *Mantella madagascariensis *group. This study strongly confirmed the existence of haplotype sharing between *M. milotympanum *and *M. crocea *and gave new insights into the phylogenetic relationships of the genus *Mantella*. Sequences of cytochrome *b *were also used to study the phylogeography of *M. bernhardi *and revealed two distinct clades corresponding to geographically isolated populations [22].

Besides the *Mantella madagascariensis *and *M. bernhardi *groups, phylogeographic studies have also been carried out on the *M. cowani *group [23]. This group is likely to be monophyletic [19,20] and includes five species according to Vences et al. (1999) [15]: *M. cowani*, which is listed in the IUCN Red List as critically endangered due to small range distribution and anthropogenic pressure [5,14,15], *M. baroni *and *M. nigricans*, both non threatened species, *M. haraldmeieri*, a vulnerable species from the extreme south-east, and *Mantella *sp. aff. *baroni *from the south-eastern Andringitra region which has been recognized as possibly distinct form by Vences et al. [15] but not formally named. Members of the *M. cowani *group are characterized by light (mostly yellow or red) flank blotches of variable extension (also found in *M. madagascariensis *group and *M. bernhardi*) and single click calls (exclusive to this group).

Cytochrome *b*-based phylogeographic data by Chiari et al. [23] were limited to two of these five species: *Mantella cowani *and *M. baroni*. Sequences of these two species formed separate haplotype networks, with haplotype sharing at one locality of their sympatric occurrence. Most intriguing, and strongly deviating from patterns found for instance in *Mantella bernhardi *[22], within both mitochondrial networks, specimens from different localities shared identical haplotypes, even those from the most distant sample sites of *M. baroni; *although most populations were characterized by a rather high haplotype diversity, no haplotype clades exclusive to geographical regions were observed.

**Figure 1:**
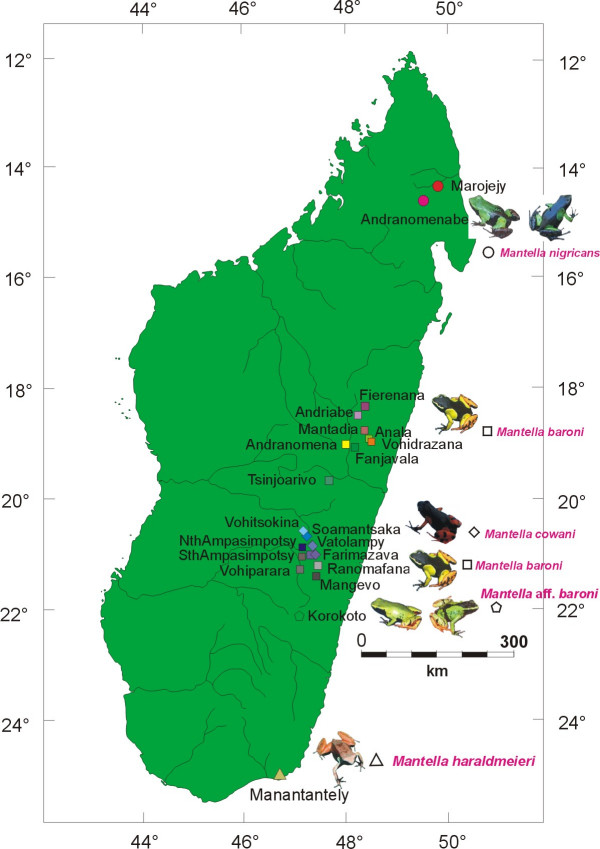
**Map of the sites and the species studied**. Colour codes of the localities correspond to those used in figure 2. The localities samples span most of the known distribution areas of all species of the *M. cowani *group, except a few additional and probably isolated highland sites for *M. cowani*, and localities of *M. nigricans *on Masoala Peninsula and on the Tsaratanana Massif.

In the present study, we provide a phylogeographic analyses that extends to all five species of the *Mantella cowani *group as recognized by Vences et al. [15]. We provide the first population-level molecular data of *Mantella *sp. aff. *baroni, M. haraldmeieri *and *M. nigricans*, and add numerous additional specimens and localities for *M. baroni *and *M. cowani *(figure 1; Table 1). Our results corroborate the status of *M. cowani *and *M. haraldmeieri *as distinct evolutionary lineages. However, the new data provide no evidence for differentiation among *M. baroni, M*. sp. aff. *baroni *and *M. nigricans *and no genetic signature of geographic differentiation within these forms, providing the first evidence for an amphibian with ongoing or recent gene flow between populations occurring across most of Madagascar's eastern rainforests.

**Table 1:** Coordinates, species and sample size for each locality

**Locality**	**Coordinates**	**Altitude**	**Species**	**Sample size**
Marojejy	14°25.948'S–49°45.583'E	671 m	*M. nigricans*	11
Andranomenabe	14°44.543'S–49°29.574'E	816 m	*M. nigricans*	5
Fierenana*	18°32'36'' S–48°26'56'' E	948 m	*M. baroni*	1*
Andriabe*	18°36'46'' S–48°19'34'' E	1047 m	*M. baroni*	5*
Mantady*	18°49'48'' S–48°25'56'' E	966 m	*M. baroni*	1*
Vohidrazana*	18°57'57'' S–48°30'37'' E	731 m	*M. baroni*	10*
Anala	18°55.142' S–48°29.257' E	813 m	*M. baroni*	2
Andranomena*	19°01'30'' S–48°10'0'' E	921 m	*M. baroni*	2*
Fanjavala	19°04.019' S–48°17.686' E	974 m	*M. baroni*	7
Tsinjoarivo*	No precise coordinates		*M. baroni*	3*
Vohitsokina*	20°42'18'' S–47°17'14'' E	1580–1620 m	*M. cowani*	20*
Soamantsaka*	20°45'22'' S–47°17'38'' E	1600–1650 m	*M. cowani*	4*
Vatolampy*	20°49'40'' S–47°19'08'' E	1540–1580 m	*M. cowani*	6*
North Ampasimpotsy	20°50'02.4'' S–47°19'59.5'' E	1332 m	*M. baroni*	8
Farimazava*	20°50'06'' S–47°19'59'' E	1380–1420 m	*M. baroni-M. cowani cowani-*	33*+11(8*+3)
South Ampasimpotsy	20°50'08.2'' S–47°19'57.6'' E	1331 m	*M. baroni*	10
Vohiparara	21°15'27.5'' S–47°21'41.5'' E	1190 m	*M. baroni*	30
Ranomafana*	21°13'34'' S–47°22'10'' E	1152 m	*M. baroni*	13*
Mangevo	21°23'14.8'' S–47°27'22.8'' E	501 m	*M. baroni*	2
Korokoto	22°11'45.4'' S–47°01'55.3'' E	848 m	*M. aff baroni*	5
Manantantely	24°59'15.2'' S–46°55'35.4'' E	81 m	*Mantella haraldmeieri*	6

Locality and sequences marked by an asterisk correspond to those already published by Chiari et al. (2005), taken and added to our dataset from Genbank.

## Results

**Figure 2:**
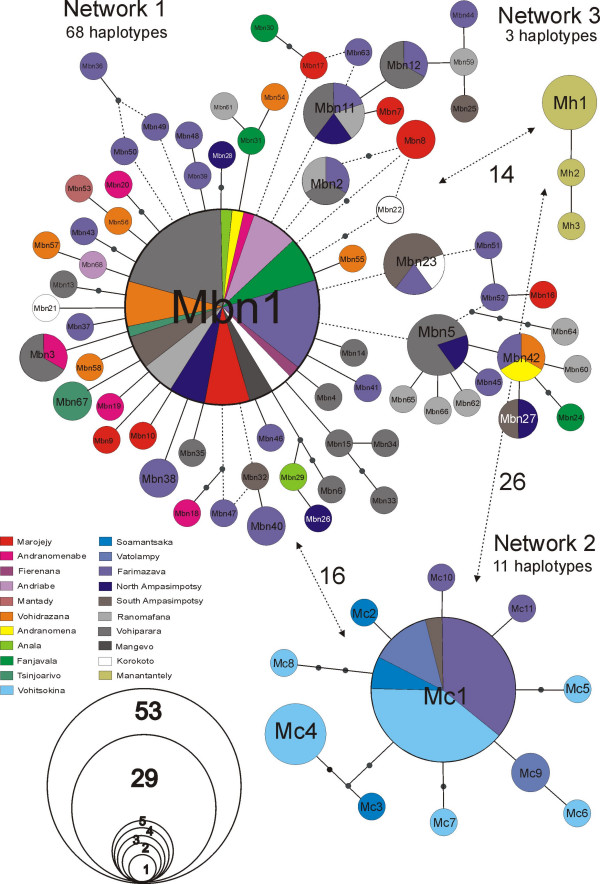
**Haplotype networks for the *Mantella cowani *group**. Mbn represents the code for haplotypes in the *Mantella baroni/M. nigricans *network, Mc represents haplotypes in the *Mantella cowani *network, and Mh represents haplotypes in the *Mantella haraldmeieri *network. Colour codes correspond to those used for the localities in Fig. 1. Dashed lines show alternative connections that were not unambiguously resolved by probability of parsimony analysis in TCS.

The total dataset consisted of 195 individual sequences of 453 bp that were distributed among 82 distinct haplotypes (of which 70 unique to single individuals) divided into three separated haplotype networks (figure 2). One network with 68 haplotypes (Mbn1 – Mbn68) from a total of 145 individuals includes sequences of *M. nigricans, M. baroni*, and *M*. aff. *baroni*, plus three putative hybrids between *M. baroni *and *M. cowani*. The second network includes eleven haplotypes (Mc1 – Mc11) from 44 individuals belonging to *Mantella cowani*, and from five putative hybrids between *M. baroni *and *M. cowani*. The third haplotype network includes three haplotypes (Mh1–Mh3) from six individuals, all belonging to *Mantella haraldmeieri*. Connecting the separate networks required distances of, respectively, 14, 16 and 26 steps between the first and second, first and third, and second and third networks. The *M. baroni-nigricans *network shows one common haplotype (Mbn1; frequency = 36%) including specimens of *M. baroni *and *M. nigricans *from all localities except Mantady (Table 2). Two individuals of *M. cowani *(two "putative hybrids") from Farimazava were also included in this haplotype network differing from the most common haplotype (Mbn1) by one and two mutations. Three out of seven individuals of *M. nigricans *from Marojejy and one out of five of *M. nigricans *from Andranomenabe share the common haplotype Mbn1. The maximum distance of *M. nigricans *noticed from this common haplotype is four mutations. The second network includes individuals of *M. cowani *(and putative hybrids between this species and *M. baroni*) with one common haplotype (Mc1; frequency = 65%). The individuals of *M. cowani *included in this network were representative of all the sampled localities, while it also contains one specimen initially identified as *M. baroni *from South-Ampasimpotsy and three *M. baroni *from Farimazava. Hence, these four individuals of *M. baroni *are to be considered as *a posteriori *hybrids (cf. definitions in [23]). The *M. haraldmeieri *(mh) network is separated from all other species and no indication for hybridization of this with any other species was detected.

**Table 2:** Distribution of haplotypes per locality after TCS analyses

**Locality**	**Sample size**	**Species**	**Number of Haplotypes**	**Common haplotypes**	**Haplotypes unique for the site**
Marojejy	11	*M. nigricans*	7	Mbn1	6 unique haplotypes
Andranomenabe	5	*M. nigricans*	5	Mbn1, Mbn3	3 unique haplotypes
Fierenana	1	*M. baroni*	1	Mbn1	0 unique haplotype
Andriabe	5	*M. baroni*	2	Mbn1	1 unique haplotype
Mantady	1	*M. baroni*	1	0	1 unique haplotype
Vohidrazana	10	*M. baroni*	7	Mbn1, Mbn42	5 unique haplotypes
Andranomena	2	*M. baroni*	2	Mbn1	1 unique haplotype
Anala	2	*M. baroni*	2	Mbn1	1 unique haplotype
Fanjavala	7	*M. baroni*	4	Mbn1	3 unique haplotypes
Tsinjoarivo	3	*M. baroni*	2	Mbn1	0 unique haplotype 1 non-unique haplotype (Mbn67 = 2ind)
Vohitsokina	20	*M. cowani*	6	Mc1	4 unique haplotypes and 1 non-unique haplotype (Mc4 = 5)
Soamantsaka	4	*M. cowani*	3	Mc1	2 unique haplotypes
Vatolampy	6	*M. cowani*	2	Mc1	1 non-unique haplotype (Mc9 = 2ind)
Farimazava	44	*M. baroni(31) M. cowani(7 pure species + 5 putative hybrids)*	25	Mbn1 Mbn2, Mbn11, Mbn12, Mbn23, Mbn42, Mc1	Mb: 15 unique haplotypes and 2 non-unique haplotypes (Mbn38 = 2 ind; Mbn40 = 2 ind) Mc: 2 unique haplotypes
North Ampasimpotsy	8	*M. baroni*	6	Mbn1, Mbn5, Mbn27	3 unique haplotypes
South Ampasimpotsy	10	*M. baroni*	6	Mbn1, Mbn23, Mbn27	3 unique haplotypes
Vohiparara	30	*M. baroni*	14	Mbn1, Mbn2, Mbn3, Mbn5, Mbn11, Mbn12	8 unique haplotypes
Ranomafana	13	*M. baroni*	11	Mbn1, Mbn2, Mbn11	8 unique haplotypes
Mangevo	2	*M. baroni*	1	Mbn1	0 unique haplotype
Korokoto	5	*M. aff. baroni*	4	Mbn1, Mbn23	2 unique haplotypes
Manantantely	6	*M. haraldmeieri*	3	Mh1	2 unique haplotypes

Mbn1 (in the *M. baroni/M. nigricans *network) and Mc1 (in the *M. cowani *network) are the most common haplotypes distributed in almost all the localities except Mantady (with just one unique haplotype collected). ind = individuals. Mc = *Mantella cowani*. Mb = *Mantella baroni*. Unique haplotypes are defined as those haplotypes present in single individuals only.

The partitioned and non-partitioned Bayesian analyses gave a consensus tree in which three clades were recovered (not shown). One internally paraphyletic clade, supported by 36% posterior probability for the non partitioned analyses and 72% for the partitioned analyses, included individuals of *Mantella baroni, M*. aff. *baroni, M. cowani/baroni *hybrids, and *Mantella nigricans*, with internal nodes supported by low posterior probability. Monophyletic groups containing all specimens of *Mantella cowani *and *M. haraldmeieri*, respectively, were recovered (with some hybrid specimens of *M. baroni *in the *M. cowani *clade) as monophyletic groups supported by 100% posterior probabilities, but these clades were nested within a clade containing sequences of *M. baroni and M. nigricans*.

## Discussion

The cytochrome *b *marker has been shown to be a good marker to identify species and hybridization phenomena [24] and to highlight genetically isolated populations [22]. Our results using the cytochrome *b *marker confirm the results of Chiari et al. [23] on the probable hybridization between *M. cowani *and *M. baroni *which, however, in general appear to be well separated species by morphology and ecology [15,23], with an uncorrected pairwise distance of about 3.5% among most of their cytochrome *b *haplotypes, and a clustering of these haplotypes in two unconnected networks. This distance appears to be in a similar order of magnitude as that between other closely related *Mantella *species, e.g., *M. aurantiaca, M. crocea *and *M. madagascariensis *(4.5–5.3%[20]).

However, the situation may be different in the case of *M. baroni, M*. sp. aff. *baroni *from Andringitra, and *M. nigricans*. The cytochrome *b *sequences used here were unable to provide any indication of genetic differentiation between these three forms, which indeed all share their most common haplotype. The structure of network 1 in figure 2 suggests that there is a high level of gene flow between these three forms. An example is offered by specimens of *M. nigricans *from Andranomenabe and *M. baroni *from Vohiparara, which shared one haplotype (Mbn3; see Table 2 and figure 2), in addition to the common haplotype Mbn1 that is present in all localities in these taxa.

The haplotypes sequenced from individuals of *M*. aff. *baroni *from Andringitra (Korokoto) do not present any significant differentiation from the most common haplotype Mbn1 in the *M. baroni/nigricans *network (maximum 1 mutation). This indicates that despite the larger extent of yellow colour in these specimens [15], they are not likely to represent a distinct taxonomical unit.

*Mantella baroni *and *M. nigricans *show very constant differences in colour pattern, such as the total absence of red or orange colour on the hind limbs of *M. nigricans*. The localities of these two taxa that were sampled in the present study are totally separated (with a minimum distance of more than 400 km from each other) which excludes a scenario of occasional hybridization. The occurrence of specimens possibly intermediate between *M. nigricans *and *M. baroni *from Folohy and from the Zahamena Reserve, within the sampling gap of our study, was mentioned by Vences et al. [15], but the available specimens from voucher collections had largely faded colour patterns and could not be attributed to either species with certainty. The contact zone of these two forms could be around these sites but more sampling in the vast area between Masoala and Moramanga is suggested to better resolve this question.

However, in general terms, the absence of a geographical structure in the haplotype differentiation of the *M. baroni/M. nigricans *complex over its distribution area indicates that these frogs have colonized their entire range very recently, and/or that gene flow between their populations is an ongoing or at least very recent phenomenon. In turn, the data would favour the hypothesis that chromatic differences between *M. nigricans *and *M. baroni *may have evolved by disruptive selective pressures and not just by genetic drift in the context of historical barriers to gene flow between a northern and southern population group, although such barriers may have evaded the resolution of our mitochondrial analyses if they had been too recent (post-Pleistocene) in age.

Taxonomically, our results suggest that *M. nigricans *may be best seen as the northern colour morph of *M. baroni*, similar to *Mantella crocea *and *M. milotympanum *[21]. Indeed, in *Mantella nigricans *and *M*. sp. aff. *baroni*, a certain chromatic differentiation between individuals of the same population (mainly regarding the extent of brown, green or yellowish colour on the dorsum) is also observed [15], confirming that in some cases conspecific *Mantella *specimens may bear different colour patterns. However, we propose not to formalize these taxonomic changes before they are confirmed by analyses of nuclear markers, and before a more stable and complete framework of *Mantella *systematics can emerge from a comprehensive analyses.

In terms of conservation, *M. baroni, M*. sp. aff. *baroni *and *M. nigricans *could be seen as a single unit of conservation based on the mitochondrial marker we used. However, it is necessary to consider the chromatic differences (of possible adaptive value) before issuing precise recommendations. In fact, different populations (or in this case different colour forms) within a species could justify specific conservation measures to preserve their genetic, ecological, and/or morphological diversity. Different definitions of conservation units have been proposed according to the parameters used to define them. From an initially broader concept of evolutionary significant unit (ESU) including ecological and genetic data, a more molecular based concept is currently mostly used (see Box 1 in [25]). However, since no ESU concepts can universally be applied, a more comprehensive adaptive evolutionary conservation (AEC) concept has been proposed [26]. The aim of the AEC concept is to preserve the evolutionary potential, thus the adaptive variance within a species (indicated under the AEC concept as ecological and or morphological as well as genetic differences) [26], which might be applicable to the three forms currently defined as *M. baroni, M. nigricans *and *M*. aff. *baroni *(from Andringitra).

The hybridization mentioned in Chiari et al. [23] between *M. baroni *and *M. cowani *is confirmed by our study, with a slightly shorter cytochrome *b *fragment but using more samples. Hybridization is a recognized phenomenon in amphibians as mentioned by numerous authors [27-29]. However, the use of nuclear marker is necessary to clearly assess the presence of hybridization between the two above mentioned species and exclude the alternative scenario of ancient haplotype sharing by incomplete lineage sorting. The present study extends records of hybridization between these two species to the locality of South Ampasimpotsy, very close to Farimazava where this phenomenon has been recorded before [23]. At these sites, the hybridization detectable at the mitochondrial level appears to affect up to 10% of the population of *M. cowani*. As observed by Andreone et al. [5], *M. cowani *deserves a special attention in terms of conservation strategy. Before 2004 this species provided a non negligible income for the pet trade [5,16]. For this purpose, we suggest: (1) *in situ *breeding programs, where parts of the original habitat are protected and promoted to stabilize the populations and in the long term possibly allow a sustainable harvesting, as described by Vines et al. [28] and (2) the constitution of new protected areas as mentioned in Chiari et al. [23].

The isolated network 3 shown in figure 2, with distances of respectively 14 and 26 mutations to *M. baroni/nigricans *and *M. cowani*, confirms that *M. haraldmeieri *is a separated unit both for considerations of taxonomy and conservation.

In conclusion, the cytochrome *b *data presented here, referring to a mitochondrial gene inherited maternally, alone do not provide a fully conclusive resolution of the taxonomy and evolutionary history of the *Mantella cowani *group. However, several important new conclusions can be drawn: (a) In the group, there are three main lineages, corresponding to *M. cowani, M. haraldmeieri*, and *M. baroni/*aff. *baroni/nigricans*, and probably these three lineages should be considered each as species in a future revised classification, (b) in the *M. baroni/*aff. *baroni/nigricans *lineage, ongoing or recent gene flow, or a very recent colonization history, led to the absence of geographic structure in the haplotype distributions, with the most common haplotype Mbn1 being universally present in populations that are almost 1000 km apart. Future studies should focus on the inclusion of nuclear markers and on closing the geographic gaps between the populations of Anjanaharibe and Fierenana, and of Korokoto and Manantantely, to be meaningfully able to apply formal tests of phylogeographic scenarios.

## Methods

### Sampling

Samples were collected from July 2003 to April 2004 at eleven localities and included all the four nominal species of the *Mantella cowani *group as well as *M*. sp. aff. *baroni *from Andringitra as defined by Vences et al. [15]. Tissues of many individuals were obtained by toe clipping, a method that is known to allow high survival rates for the released individuals (>98%) [30]. Samples were preserved in 99% ethanol. Representative specimens from the new localities were collected and preserved as voucher specimen in the collection of the Département de Biologie Animale of the Faculty of Sciences of Antananarivo, partly after skinning them for analyses of alkaloids; from these specimens, larger tissue samples of femur muscle were taken. The newly collected samples (season 2003–2004) came from two localities of *M. nigricans*, one locality of *M. haraldmeieri*, seven localities of *Mantella baroni *and one locality where *M. baroni and M. cowani *live in sympatry (Table 1). Among the new collecting sites, only one (Farimazava) had already been sampled before [23]; three additional sequences of *M. cowani *are here added from this locality.

The new sequences were combined with sequences from a previous study [23] corresponding to individuals from twelve populations: four populations of *M. cowani *and eight populations of *M. baroni *with the one locality (Farimazava) of sympatry. As in Chiari et al. [23], two putative hybrids between *M. cowani *and *M. baroni *were included in our study, defined as individuals with an orange-yellowish coloration, more extended lateral spots (versus small and rounded spots in *M. cowani*), residual of cephalic lines (clearly delimited in *M. baroni *and lacking in *M. cowani*) and presence of yellowish shading on tibiae (versus red bands in *M. cowani *and black-orange patterned tibiae in *M. baroni*. Table 1 summarizes the localities, the species and the sample size of each species used in this study. The map of the localities of each species is presented in figure 1.

### Laboratory techniques

Total genomic DNA was extracted from the tissue samples or toe clips using proteinase K digestion (1 mg/ml concentration) followed by a standard salt extraction protocol [31]. We used the primers Cytb-c 5'-CTACTGGTTGTCCTCCGATTCATGT-3' (forward) and CBJ10933 5'-TATGTTCTACCATGAGGACAAATATC-3' (reverse) [32] to amplify one part of the mitochondrial cytochrome *b *gene of about 600 bp. PCRs were performed in 25.6 μl reactions using 1 μl of genomic DNA, 0.8 μl of each 10 μmol primer, 2 μl of total dNTP 4 mM, 1 μl additional 25 mmol MgCl_2_, 2.5 μl 10× NH4 superTaq PCR buffer (HT Biotechnology), 0.25 μl of 5 u/μl iTaq DNA Polymerase (HT Biotechnology) and 17.25 μl of water. PCR conditions were performed with an initial denaturation step at 94°C for 90 seconds; 35 cycles at 94°C for 30 seconds, annealing temperature of 53°C for 45 seconds followed by 30 seconds at 72°C; and a final extension of 10:00 min at 72°C. PCR products were loaded on 1% agarose gels, stained with ethidium bromide, and visualised on a "Gel Doc" system (Syngene). Products were purified using QIAquick spin columns (Qiagen) prior to cycle sequencing. A 10 μl sequencing reaction included 1 μl of template, 1.75 μl of 5× sequencing buffer (BigDye Terminator Sequencing buffer, Applied Biosystems), 1 μl of 10 μmol primer (Cytb-c or CBJ), 0.5 μl of ABI sequence mix (BigDye Terminator V1.1 Sequencing Standard, Applied Biosystems) and 5.75 μl of water. The sequence reaction was performed as follows: 3:00 min preheating at 90°C, 24 cycles of 30 seconds at 96°C, 15 seconds at 50°C and 4:00 min at 60°C, final extension 4 min at 60°C. Sequence data collection and visualisation were performed on an ABI 3100 or an ABI 3730 automated sequencer at the sequencing facility of the Medical Center of the University of Amsterdam.

### Analysis techniques

The sequences were checked and aligned using the software Sequence Navigator (Applied Biosystems). Sequences were deposited in GenBank (accession numbers DQ889341–DQ889429). The program Collapse v3.1 [33] was used to merge sequences into haplotypes. Modeltest version 3.7 [34,35] was used in conjunction with PAUP*, version 4.0b10 [37] to estimate the best fitting models for our complete dataset and for each partition corresponding to the different codon positions of the cytochrome *b *gene. Based on the Akaike Information Criterion (AIC), the following models of sequence evolution were determined: (1) for the first codon positions, a TrNef model with a proportion of invariable sites of 0 and equal rates for all sites; (2) for the second codon positions, a F81 model with a proportion of invariable sites of 0 and equal rates for all sites; (3) for the third codon position, a GTR model with a proportion of invariable sites of 0 and equal rates for all sites; (4) for the complete dataset, a GTR model with a proportion of invariable sites of 0 and variable sites distributed according to a gamma distribution shape parameter of 0.3438. Bayesian phylogenetic inference using the program MrBayes 3.1.1. [36] was carried out with substitution settings adjusted according to these substitution models, and with four chains with the default heating values, 1,000,000 generations, saving the tree at every tenth generation and discarding the initial 10,000 trees as burn-in. We ran both partitioned and non partitioned analyses, both of which yielded almost identical results. *Mantella aurantiaca *was used as the outgroup.

Haplotype networks were constructed using the TCS software package [38], which employs the method of Templeton et al. [39]. It calculates the number of mutational steps by which pairwise haplotypes differ and computes the probability of parsimony [39] for pairwise differences until the probability exceeds 0.95.

## Competing interests

The author(s) declare that they have no competing interests.

## Authors' contributions

FCER carried out the data collection, the molecular genetic laboratory works, participated in the sequence alignment and analyses and drafted the first manuscript. YC participated in the sequence alignment, conceived of the analyses and interpretation of data and participated in the critical revision of the manuscript. ORR conceived of the study, participated in the data collection, made a part of laboratory work and the conception of the manuscript. MV participated in the design and coordination of all the work and participated critically in the writing. All authors read and approved the final manuscript.
